# Gerbils without Borders: Invasiveness, Plague, and Micro-Global Histories of Science, 1932-1939

**DOI:** 10.1093/jhmas/jrae041

**Published:** 2025-10-08

**Authors:** Jules Skotnes-Brown, Matheus Alves Duarte da Silva

**Affiliations:** University of St Andrews, Scotland, UK

**Keywords:** species invasiveness, bubonic plague, gerbils, animal history, microhistory, global history, history of science, Baixo Cunene, Ovamboland

## Abstract

In the 1930s, a series of bubonic plague outbreaks among humans cropped up in several villages at the border of Angola and Namibia. These outbreaks provoked deep concern, laying bare social and political tensions amongst neighboring imperial powers and Indigenous people within the region. Despite the appearance of this disease in what was then considered a recondite place, its spread sparked debate in transnational forums, such as the League of Nations and the *Office International d’Hygiène Publique*. Drawing upon archival records in Namibia, South Africa, Portugal, the United States, and the United Kingdom, this article argues that concerns over the spread of plague across land borders led to the development of a nascent invasive species framework which indicted border-crossing “migrant” South African gerbils for the international spread of the disease. It follows the transnational political and scientific dynamics created by the plague “invasion” and discusses how these, like the gerbils, crossed numerous borders and scales. Ultimately, this article shows how localized inter-species and inter-imperial encounters can provide empirical insights into the feasibilities of a micro-global history of science in which more-than-human actors take on an important role.

In early January 1932, Michiel van Niekerk, the District Surgeon of Ovamboland, South West Africa (present-day Namibia) noticed an unexpected series of bubonic plague symptoms in Indigenous Ovambo patients. Several cases of “glandular swellings in the groin” of patients had appeared in the region, but since the symptoms were mild, he thought little of it.^1^ Plague seemed an incredibly unlikely prospect so far north and so distant from the coast.^2^ However by the end of January, smears were taken from the patients, and *Yersinia pestis*, the causative agent of plague, was found.^3^ Shortly afterwards, evidence of the bacterium was detected in the carcass of an “apparently healthy” gerbil that van Niekerk dug up near his house.^4^ By 8 February a cluster of twenty-five human cases were discovered in the Ondonga region, Ovamboland, with five deaths. This cluster slowly grew, and the disease spread from its epicenter at Ondonga, to Oukwanyama, Ombalantu, Ongandjera, Uukwaluudhi, Uukwambi and finally Onkolonkathi by the end of 1932 (see Figure 1).^5^ Already in February 1932, the discovery of plague in Ovamboland sparked concerns in Angola over the risks that it could soon cross the border into the Baixo Cunene region.^6^ By the end of 1932, these concerns became reality, with the Baixo Cunene registering its first two cases and subsequently nine human deaths in the following year.^7^ After 1937, plague cases among humans and rodents disappeared from the border.^8^

From an epidemiological point of view, plague morbidity and mortality were quite low – the Ovamboland outbreak of 1932, the biggest in the area, claimed the lives of fifty-six people – yet the arrival of the disease was regarded as a political and medical crisis on local, colonial, and even global levels.^9^ This rural border region became a staging ground for major scientific and diplomatic developments of the 1930s. Investigations conducted by South West African, South African, and Portuguese scientists, in collaboration with local settlers and Indigenous people, revealed that what South Africans called “veld plague” (once a localized disease specific to rural South Africa), or what the Portuguese doctor Ricardo Jorge had coined *sylvatic plague* in 1927 (plague among wild rodents living in circumscribed uninhabited spaces) could silently transgress international borders and that scientific collaboration, rather than inter-imperial hostility and Indigenous repression, promised its solution.^10^ Such research also led to the framing of Southern African gerbils as invasive species, not because they were detrimental to indigenous fauna, but because of the risks they posed to public health. In Southern Africa, the idea of gerbils as invasive arose primarily from medical research and concerns over human health that had framed plague and its causative agent, *Y. pestis*, as invasive, and from collaboration between doctors with Indigenous people albeit under highly unequal power structures. This demonstrates a largely neglected angle in the historiography of the idea of invasive species: that in the 1930s, concerns over human health were central to the idea of certain animals as invasive.

By focusing on the agency of gerbils to cross borders, this article shows the importance of incorporating animals as actors into the history of science, medicine, and global history. All human actors in this story – whether Portuguese, Angolan, South West African, South Africans, or representatives of international organizations – were brought together because of the perceived agency of Southern African gerbils in migrating across colonial borders. Therefore, this article also contributes to ongoing dialogues among microhistory, the history of science, medicine, and global history. Although these frameworks have recently been brought into conversation, most “global micro-history of science” has stressed the movement of human actors, the objects they circulated (such as maps, seeds, and vaccines), and the new knowledge that was produced in this process.^11^ This article highlights a connected question, discussing how peoples’ conceptions of the transnational movements of gerbils independently of humans and human-made infrastructures pushed for new nascent ecological explanations of disease in Southern Africa, which were intimately connected with discourses of invasion. Here, we are less concerned with documenting exactly what these gerbils were doing in the past and more with how various human actors conceptualized, theorized, and acted against their movements, habits, and settlement practices, leaving traces of their agency in colonial texts in the process.^12^ In other words, although it is impossible for us to know for certain whether gerbils were the primary agents in spreading plague across the border, their perceived agency in doing so produced tangible consequences. Indeed, it was this perceived agency, and especially what were understood to be the interrelations among gerbils, microorganisms, dune ecologies, transport infrastructures, groups of humans, and sources of food, which brought diverse groups of human actors together and justified a series of sanitary actions. Gerbils thus play a key role in this story as actors around which medical and ecological knowledge was constructed and sanitary practices were developed.

## Ovamboland: Colonial Politics and Plague, 1920S-1930S

**Figure 2:**
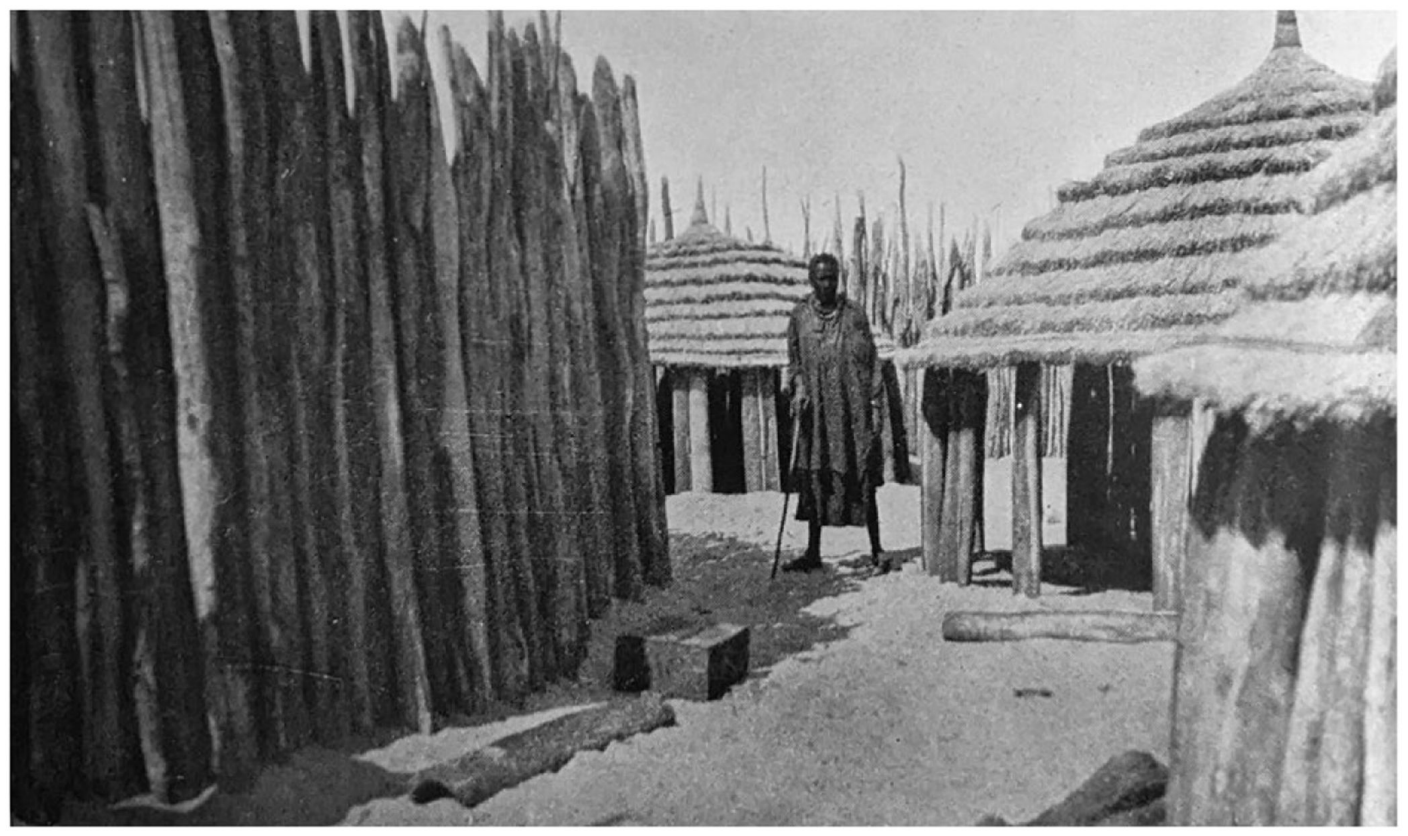
A view of the interior of an *eumbo*. The original caption reads: “Interior of a headman’s kraal. A section of his personal quarters.” From C.H.L. Hahn, L. Fourie and H. Vedder, *The Native Tribes of South West Africa* (Cape Town: Cape Times Limited, 1928), insert at page 8.

The environmental and political history of Ovamboland in the 1930s is complex, and closely entangled with the outbreak of plague and its control. The region was partially within the boundaries of South West Africa (henceforth SWA), but never incorporated into its White-controlled “police zone.” After World War I, Germany lost its African colonies, and, along with the rest of SWA, Ovamboland became the responsibility of South Africa under its Class C League of Nations Mandate over the former German colony, which rendered it a colony of South Africa in all but name.^13^ By the 1930s, permanent White settlement in Ovamboland was forbidden, with exception of political officials who administered it under indirect rule. The region was governed in collaboration with so-called Chiefs and Headmen who held some administrative autonomy over “tribes.”^14^ Ovamboland was a populous region with more than 150,000 residents.^15^ By comparison, the population of the capital of SWA, Windhoek, was only 10,651 in 1936.^16^ Most people lived within roughly circular homesteads called *eumbos* which included individual huts, cattle enclosures, as well as crop and grain storage areas (see Figure 2).^17^ The people within this region were designated “Ovambo,” which homogenized a diversity of groups and languages. Ovambo nevertheless remains in use as a shorthand for the diverse political groupings who lived in this region.^18^

Ovamboland was also the site of a long-contested border between SWA and Angola, and a “Neutral Zone” between these territories, which was progressively delimited and eventually handed over to Angola in 1928.^19^ Right up until the outbreak of plague, this border was porous to the movement of Ovambo people, especially the Oukwanyama who had lived in the Neutral Zone. Some Ovambo leaders had subjects on either side, while others moved between them in response to repressive colonial policies, ecological pressures, or the pursuit of wealth.^20^ The Oukwanyama in Namibia, as well as all Ovambo peoples in southern Angola, spoke Oshikwanyama, indicating long linguistic and cultural ties across the border.^21^

In the 1910s-1920s, the region experienced a series of colonial-caused crises: Portuguese and German warmongering, attempts to destabilize Ovambo political power by deposing powerful leaders, South African migrant labor recruiting, and most-importantly, two major famines.^22^ In 1932, Ovamboland remained a politically unstable area in which the South African colonial state was particularly weak. The Ukwambi and Oukwanyama resisted the authority of Native Commissioner Carl “Cocky” Hahn, and after a heated standoff between Ukwambi King Iipumbu yaShilongo and a group of missionaries in the region, the South African army staged a gruesome display of military power by bombing Iipumbu yaShilongo’s compound with airplanes and banishing him.^23^ The outbreak of plague was thus the latest in a litany of problems that occupied colonial administrators in this region, and given the severity of the disease, one that needed to be tackled swiftly and decisively.

Initially, van Niekerk blamed what he regarded as Ovambo diets for the outbreak, writing that “the country is simply overrun with mice and other rodents which form a liberal part of the native diet.”^24^ Medical Officer F.C.S. Hinsbeeck similarly insisted that Ovambo people “dispense with that article of diet” and “carry out an active campaign against rodents living in the vicinity of their habitations.”^25^ Just a few weeks later, van Niekerk was relieved to hear that “Chief Martin” Nambala yaKadhikwa had told him that since “considerable numbers of dead rats have been found in their burrows,” this had “scared the natives off their mouse diet.”^26^ Although contact between Ovambo people and rodents helped explain the incidence of the disease to colonial officials, it did little to demonstrate how plague had arrived in Ovamboland in the first place. After all, this disease had not even been detected in the major cities or ports of SWA.^27^ The idea of nonhuman invasions was first invoked here in response not only to animals, but also to microorganisms. The notion of *Y. pestis* as an invasive microorganism carried in the bodies of migratory people and animals shaped the rhetorical foundations of three competing theories about the emergence and epidemiology of the disease.

## Initial Theories of the Ovamboland Plague: South African Grain and Ovambo People

In a 1932 letter to Edward Thornton, Secretary of Public Health of South Africa, South West African Medical Officer F.C.S. Hinsbeeck offered one theory on the matter. Based on a long-standing colonial belief that plague could spread in bales of grain, he argued that cereals from the Union of South Africa, transported for famine relief in the 1930s, had “directly imported” plague to Ovamboland and that the agency of rodents in response to famine had spread it from place to place.^28^ He wrote that mealie meal and corn,

was sent to Ondongua [sic, present day Ondonga] from Pietersburg and I think also from the Orange Free State, in which latter area plague is endemic…Ondongua is situated practically in the centre of Ovamboland and the outbreak has been confined to the area in the immediate vicinity of that station, which was the distributing centre. The rodents would have so to speak concentrated on that centre as “veld-kos” [foraged food] was not available owing to the severe drought.^29^

Hinsbeeck knew this was a controversial claim to make. Although rodents shared some of the blame, he was accusing the South African government of indirectly exporting plague within the bodies of rodents or fleas, nestled in bags of grain, and he added that he hoped that it “cannot be substantiated.”^30^ Claiming that South Africa was exporting plague had broader ramifications than a small outbreak in Ovamboland: it meant that “other infected grain must have been leaving the Union.”^31^ This theory risked undermining the reputation of South African produce and causing serious economic and political damage. It also risked discrediting South Africa’s commitment to the welfare of the region. Public health was regarded as a major responsibility of the Mandate Administration and a potential target of critique from African nationalists. Three years previously, former District Surgeon of Ovamboland Johannes Hendrik Loots had worried that healthcare in the region left much to be desired, and that “paid agitators with Bolshevik tendencies” might “point out the obvious deterioration and disintegration of the native race, under European protection.”^32^ The South African delegate to the League of Nations evidently recognized these risks, writing that the grain theory might make “a bad impression on the Mandates Commission,” which risked demonstrating South Africa’s ineptitude in governing SWA.^33^

Despite initially depicting the plague bacillus as an organism which had passively been “directly imported” into the region, by April Hinsbeeck thought that plague had acquired an active and invasive character. He wrote that although the disease had not yet “occurred amongst the western tribes” or “the Northern Tribe…before long rodent plague if it has not already *invaded* the areas occupied by the aforementioned tribes *will do so*.”^34^

On 20 February 1932, the Governor of the Province of Huila, on the Angolan side of the border, informed Luanda of the appearance of plague in Ovamboland.^35^ The Head of Angola Sanitary Service, Antonio Damas Mora, responded swiftly and decisively. Fearing that humans could play a role in spreading the plague, the Angolan Government authorized sending seventy “native soldiers” to the border, creating a twenty kilometer “cordon sanitaire.”^36^ Their instructions were to prevent humans and cattle from crossing the border on either side, by force if necessary.^37^ It is unlikely that Damas Mora imagined that humans could directly spread plague, except in the case of the pneumonic plague which was relatively rare in Angola. Indeed, when discussing the first outbreak of Angola in 1921, he penned that “no signs in Angola have ever led us to think that bubonic plague could have had a human origin.”^38^ Instead, Damas Mora argued that people fleeing infected places might transport rat fleas in their clothes, rags, and luggage, and thus expand the plague area.^39^

The cordon sanitaire theoretically applied to Europeans and Ovambo people alike.^40^ Nevertheless, the latter were the most affected by it, as the cordon separated families who had relatives on opposing sides of the border and prevented the free grazing of cattle in the more fertile Angolan side. As such, several violent skirmishes soon took place along the border.^41^ These clashes were particularly worrying to the Union authorities, who feared that armed conflict might arise between Angolan troops and Ovambo people, and that the Angolans were breaking international law by allowing troops to cross the border.^42^ In other words, the Portuguese officers stationed in Angola were framing SWA Ovambo people as potential plague carriers, while the South Africans feared something akin to a military invasion – the incursion of foreign troops into their territory. Seeking to diffuse tensions, the South West African authorities successfully negotiated the cessation of the Portuguese military cordon by 20 April 1932.^43^

However, in opposing the militarization of the border, South West African officials were not attempting to challenge the view of the Ovambo as facilitating the movement of plague into new areas. Like their Portuguese counterparts, South West African officials pathologized alleged Ovambo habits as unhygienic and conducive to disease. In 1929, Loots claimed, in an utterly erroneous and contemptuously racist statement,^44^ that the Ovambo “native has no idea of sanitation. He urinates and defaecates literally on his doorstep, Flies abound in millions all the year round. Large mud holes serve as water supply, which during the dry season is little more than a thick, black or greenish slimy ooze. During the rainy season human and animal excreta, dead animal and what not, are carried into these water holes.”^45^ Unsurprisingly then, South West African officials also framed Ovambo people as potential spreaders of plague, and expressed fears that their movements would enable plague to enter the White-populated police zone. Ovamboland was, at this point, an important source of migrant labor for the mines and farms in the south.^46^ The very underpinning of this economic system facilitated mass movement of people from Ovamboland into the mining towns of Tsumeb, Luderitz, and parts of the Union of South Africa. Assuming that this risked facilitating the spread of plague into new areas, South West Africans initially placed their own sanitary cordon around Ovamboland, forbidding Africans from leaving the area.^47^

Both pathologizations – of South African grain and of Ovambo people as facilitating the spread of plague into new areas – had worrying implications for Union authorities. The former risked the reputation of an important export and was considered a threat to South Africa’s mandate, while the latter was interrupting supplies of labor to the mines in SWA and the Union. At the request of Hinsbeeck, the Secretary for SWA asked the Union to send a “Medical Officer” to assist in the investigation.^48^ Their choice was Louis Fourie, in many ways, the perfect man for the job.

Fourie, a former South West African Medical Officer with a deep interest in ethnography and the co-author of *The Native Tribes of South West Africa*, was based at the South African Public Health Department and had extensive experience investigating plague in rural areas of South Africa.^49^ Fourie arrived on 31 March and set out straight to work. In his research, he visited all plague infected areas, liaising with South West African and Ovambo political authorities, as well as Portuguese doctors and diplomats.^50^ His report challenged both the grain theory and the pathologization of Ovambo people, instead shifting the blame onto Taterona gerbils (a term which now refers to ten sub-species of Southern African gerbils).^51^ Unlike Hinsbeeck, Fourie did not explicitly refer to plague as an invasion, instead indicting migratory gerbils for creating an underground network through which the disease could spread internationally.^52^ Almost certainly recognizing the deep political and economic problems that it posed, Fourie sought to dismantle the grain theory in his report. To do so, he developed his own interpretation of the agency of rodents, and in the process, exonerated South African grain while challenging the idea that Ovambo people played an important role in the spread of the disease.

Firstly, he wrote that the “abundance and luxuriant growth of grass and other seed-bearing vegetation in Ovamboland and the Kalahari region furnish a permanent and plentiful supply of food for the indigenous rodents” – the implication being that rodents were unlikely to congregate around imported bales of grain. Having been personally involved in anti-plague operations in the Orange Free State, Fourie was convinced that during 1929-1930, “no case of infection from the handling of infected grain occurred on any mills or railway stations” in that province.^53^ Also, in “no instance” in the Union was it known that “infection occurred at a distance as a result of rail carriage of infected produce.”^54^ Finally, nobody observed any live or dead rodents on route to SWA, as far as he “was able to ascertain.”^55^

Instead, Fourie placed blame for the outbreak on the alleged migratory activities of Taterona gerbils. He argued that field evidence indicated that plague infection had traversed South-eastern Ondonga “about 12 months ago” along the “main sand-dunes.”^56^ This was because there was evidence of “a wave of infection among Taterona gerbilles some time prior to the summer of 1930-31” because their burrows have been “effaced almost completely.”^57^ What had likely happened, he suggested, was that there had been a “wave of infection from the South having spread northwards through the Kalahari during 1930 and 1931.”^58^ Although this still implied the disease had arrived from the Union, it took the blame away from humans. The agency of gerbils had spread the disease, rather than commerce from the south. The geography of the area was also to blame, which enabled gerbils to create a highway across which *Y. pestis* could move: “The dune formation of the Kalahari extends uninterrupedly [sic] into Ovamboland and, being heavily infested with Taterona gerbilles is pre-eminently favourable for the rapid spread of rodent plague, which, in the Union, has been known, in some instances, to advance at the rate of 300 odd miles in one month.”^59^ To explain this, Fourie depicted the desert sand dunes as a kind of network through which a “wave of infection” could “spread northwards.”^60^ Burrow activity, he claimed, indicated that gerbils tended to migrate between sand dunes and Oshanas (riverbeds that flood and dry out) in the dry season. Plague waves would quickly decimate the dune burrows and slowly infect the more isolated Oshana gerbils “some time after the primary wave has passed.”^61^ Essentially, the desert sands and their gerbil denizens had facilitated the movement of *Y. pestis* to Ovamboland.

Fourie also reported what appeared to be evidence of a new gerbil species in Ovamboland: *Desmodillus auricularis pudicus*. This animal had a “very wide” distribution in SWA, including in Ovamboland, but was not recorded in a “mammal survey of Ovamboland” conducted by Captain Guy Shortridge of the King William’s Town Museum in the 1920s.^62^ Fourie provided no further commentary on this, but his collaborator Hinsbeeck added that *Desmodillus auricularis* was “of interest” because it was “unknown to the natives,” unlike the other rodents in the region which were “known by native names.” This “desert or Namaqua Gerbille,” he claimed, was “found in the Union,” “peculiarly marked and cannot be easily mistaken.”^63^ Hinsbeeck’s implication was clear: a foreign species had established itself in the region. A nascent invasive species framework was emerging.

In order to control the disease in Ovamboland, Fourie thus made several recommendations in a report circulated in June 1932. Since the disease was allegedly endemic throughout SWA, control policy needed to gravitate around routine surveillance, rodent-proofing, and killing of rodents in the vicinity of human settlements. This involved a suite of measures and recommendations comprising anti-plague serotherapy, vaccination, quarantining cases of pneumonic plague, supervising burials, education, and the “disinfestation” of homes, clothing, and domestic animals. Additionally, Fourie suggested attacking gerbil burrows through fumigation. Critical here was the collaboration of Ovambo leaders, who needed to be encouraged to wage relentless war against rodents in their homesteads. Children were of particular importance as they were, in Fourie’s view, already prolific rodent-catchers who caught and consumed the animals for fun. Finally, Fourie insisted on the permanent appointment of a rodent inspector, suggesting a man he trained, K. Schettler.^64^

Fourie succeeded not only in formulating a response to plague but also proved a shrewd diplomat and played a major role in averting the brewing political crisis. Migratory gerbils were the perfect target of blame. Fourie’s theory deflected attention away from the embarrassing possibility that South African grain was infected, diffused the border tensions with Angola by acquitting the Ovambo of the primary epidemic blame, and provided a strategy of repelling plague from areas of human settlement. Even Portuguese officials, who took a dim view of their South West African counterparts, were impressed by Fourie.^65^ Fourie’s report ultimately restored the damaged relationships between South West African and Angolan authorities and inaugurated a period of closer cooperation. Over time, he and his research enabled the formation of scientific networks among SWA, South Africa, Angola, and Portugal.

## Plague in Angola’s Southern Border

When plague appeared at the southern border of Angola in 1932, the disease was not unknown to Portuguese doctors. The third plague pandemic (1894-1950) had affected Portuguese colonies since the end of the nineteenth century; Macau, in China, Damão, in India, and Mozambique, on east coast of Africa, registered outbreaks between 1895-1899.^66^ Even Porto, Portugal’s second biggest city, was attacked by plague in 1899 in one of the major outbreaks in Western Europe. To quell the epidemic, Portuguese authorities established a cordon sanitaire around Porto, sparking a public outcry and international criticism.^67^ On the other hand, Portuguese Atlantic Ocean African colonies were unaffected by plague in the first two decades of the pandemic. Only in February 1921 did Angola register its first outbreak in its capital and biggest port, Luanda.^68^ In the following decade, plague primarily affected the Angolan coast and occasionally inland cities, such as Catete and Malange (see Figure 2).^69^ The idea that plague would reach the Baixo Cunene was considered almost impossible, given its great distance from the Atlantic and the main ports.^70^

The news about plague in Ovamboland was officially communicated on 20 February, sparking a controversial cordon sanitaire.^71^ On 7 May 1932 the Portuguese Consul in Windhoek, Adolpho Trindade, informed Lisbon that he had met with Hinsbeeck and Fourie. Trindade noted that both supported different theories about the spread of plague to Ovamboland and that he did not take a side on the controversy. Instead, Trindade emphasized that both theories showed the Ovamboland outbreak was not Angola’s fault, but that it meant that “plague is already close to our border and that it will soon appear in Angola.”^72^ When forwarding this letter to the Director of the Colonies and Health, Trindade’s superior, F.A. Correia, asked his counterparts whether the International Health Organizations had already discussed this matter or if they would only do so “when plague invaded also the South of Angola.”^73^ On 17 May 1932, the *Office International d’Hygiène Publique* (OIHP), one of these international health organizations mentioned by Correia, notified its members that Ovamboland was registering cases of plague among humans, that wild rodents were found dead there, and that this epizootic was “extending to the North and to the North-West.”^74^ Nonetheless, the OIHP did not establish a clear connection between these events.^75^ One month later and with a summary of Fourie’s conclusions in hand, the OIHP stated that “a speedy study enabled the conclusion that the epidemics are due to the expansion of the rodent plague that is occurring in the northern districts of the South-Kalahari toward the Ovamboland. Today, the line reached by the rodent plague is in the Ukuanyama, at the border with Angola.”^76^ An epidemic “prophecy” was forming amongst Portuguese colonialists and international health agencies: sooner or later plague might reach the Baixo Cunene, and this “invasion” or “expansion” would likely be linked to wild rodents.^77^

Amidst these concerns, the Angolan government organized a scientific commission, the *Missão Médica de Defesa e Combate contra a Peste na Fronteira Sul de Angola* (henceforth Missão).^78^ The Missão arrived in Vila Pereira de Eça (now Ondjiva), in the Baixo Cunene, on 24 June 1932.^79^ According to the first report by the Missão, the Baixo Cunene was inhabited by around 55,000 “natives,” divided by the Portuguese administration in five main “tribes,” “Ambos,” “Bangalas,” “Hereros,” “Ganguelas,” and “Quiocos,” with some of the tribes being organized around “sub-tribes.” The “Cuanhamas” (likely referring to the Oukwanyama), a sub-tribe of the “Ambós,” formed the bulk of the population of the Baixo Cunene with 33,267 individuals.^80^

The Baixo Cunene had a relatively long history within Angola and the Portuguese Empire. Portuguese explorers had traversed the Baixo Cunene/Ovamboland for centuries and since 1844 trade routes for ivory and enslaved people connected this region to other parts of the Portuguese Empire.^81^ Nevertheless, until the Berlin conference (1884-1885) the Portuguese Empire had attributed little importance to the region, concentrating its colonial presence around the coast. After the conference bequeathed SWA to Germany, Portugal slowly began the military occupation of the Baixo Cunene over fears it could later be claimed by Germany or other European imperial powers. Nonetheless, Portuguese settlement was constantly prevented by the fierce opposition of the Oukwanyama and by German troops. After the defeat of these two enemies during WWI, the Portuguese managed to take control of the Baixo Cunene, but their presence remained sparse until the 1930s.^82^

The boundaries between the German and Portuguese empires, and later the South African mandated territory, remained blurred for decades. Germany established a neutral zone of around 400 by 11 kilometers between Angola and SWA, securing its access to the Cunene and Cubango rivers. After the German defeat in WWI, Portugal engaged in talks with Britain and South Africa to settle the disputes by establishing a formal border. In 1928, the border was finally defined, the neutral zone officially abolished and incorporated by Angola. The land border split the Oukwanyama population between Ovamboland and the Baixo Cunene, and although movement over the border continued, it was more strictly regulated.^83^

The Baixo Cunene, and especially the border with SWA, was thus considered a backwater when looked at from Luanda or Lisbon, a region recently incorporated into the Portuguese Empire, and a politically tense area. The memories of the battles among Portuguese, Oukwanyama, and other local groups, as well as other geopolitical issues were vivid in the minds of the Portuguese members of the Missão. In the introduction to Missão’s first report, the Governor of Huila, Raimundo Serrão, observed that the Baixo Cunene “was to date the region [in Angola] where more of our brothers died to enemy bullets than anywhere else, and because the region was the envy of foreigners, so much ink and diplomacy are still being spent [on it].”^84^ Therefore, even if the Missão’s official goal was to address a sanitary concern, it was also a demonstration to the “natives,” to the few Portuguese in the region, and maybe more importantly, to other Empires in Africa, that the Baixo Cunene was Portuguese and that the Portuguese Empire was present there, paternalistically taking care of European and Indigenous people alike.^85^

The Missão was directed by Dr. Francisco Venâncio da Silva, assisted by Dr. Fernando Damas Mora (Antonio Damas Mora’s son) and three male nurses.^86^ Silva had already encountered plague in the Portuguese colony of Guinea in 1920 and in Catete, Angola.^87^ Soon after his arrival to the Baixo Cunene, Silva visited the South West African side of the border, was shown “typical plague cases” by van Niekerk, and watched the “disinfestation of a kraal by the Rodent Inspector and the destruction of field rodents in their burrows by means of cyanogas through pumping.”^88^ Silva was, according to Native Commissioner Hahn, “very impressed” by this, and asked if one of his medical orderlies could go to Ondangwa to “learn the latest methods of disinfestation from our Rodent Inspector, Mr Schettler,” which he agreed to, as he was “anxious to co-operate and assist.”^89^ Interactions at the border were becoming more peaceful than in the early days of the outbreak.

In its first weeks and months, the Missão concentrated on vaccinating the population against plague and in capturing and examining rats and rodents. Some of these animals showed symptoms of plague, indicating that the infection had already crossed the border.^90^ These works produced several tangible results. On a local level, Silva agreed with Fourie that the outbreak in Ovamboland was linked to wild rodents. Consequently, the Missão started deploying anti-rodent measures in the Baixo Cunene, inviting the local Portuguese settlers and Indigenous people to help by killing rodents and rodent-proofing their houses.^91^ Given the spread of the infection to wild rodents on the Angolan side of the border, in September 1932 the Portuguese government decided to transform the Missão into a long-term service, dubbed “Serviço Permanente de Prevenção e Combate à Epidemia de Peste Bubónica no Sul de Angola” (henceforth Serviço), whose task would be to fight plague mainly by deploying anti-wild rodents measures.^92^

In sum, Fourie’s theory – which blamed gerbils and proposed policing their transnational movements – placated Portuguese, South West African, and South African colonial authorities, as well as Ovambo people, and brought both sides of the border into collaboration. As a result, by the end of 1932, his report and his theory of gerbil migration were accepted by those closest to the affair with few exceptions. In fact, Fourie’s theory was still considered valid among Portuguese doctors into the early 1970s.^93^

## Globalizing the Knowledge Constructed at the Border

Despite Fourie’s successes and the perception within the SWA and South African Public Health Departments that they had brought the plague problem under control, the affair had wide-reaching transnational consequences which were aired at a League of Nations conference in Cape Town in November 1932.^94^ The event proved an embarrassing affair for South West Africans, with the delegate of Angola, Antonio Damas Mora, expressing “very strong views as to the inadequacy of the South West Plague Staff, and anxiety as to what might happen on the South West Africa – Angola border should heavy rain occur.”^95^ South African Secretary of Public Health Edward Thornton thought that such a critique meant that further South African intervention in the region might be necessary, writing that “Questions…could quite properly be raised by the League of Nations as to the duties and responsibilities of the Mandatory Power should rain occur and the disease flare up.”^96^ Moreover, delegates at the conference floated a disturbing possibility: despite all of their technological advances and international agreements, the invasion of plague was proving impossible to contain. In an official report of the proceedings, S.W.T. Lee depicted plague as an invasive disease, carried by “wild rodents,” writing that since infection had “overrun the Union borders and has invaded the Mandated Territory of S.W. Africa and also Angola,” it would likely spread across the entire sub-continent since there was no “demonstrable factor which can be expected to prevent the slow spread of plague North and East.” To contend with this threat, Lee argued that closer international cooperation in rodent and plague surveillance and control was necessary.^97^ Lee’s suggestions became, in a slightly adapted form, the official conclusions of the conference.^98^

**Figure 3:**
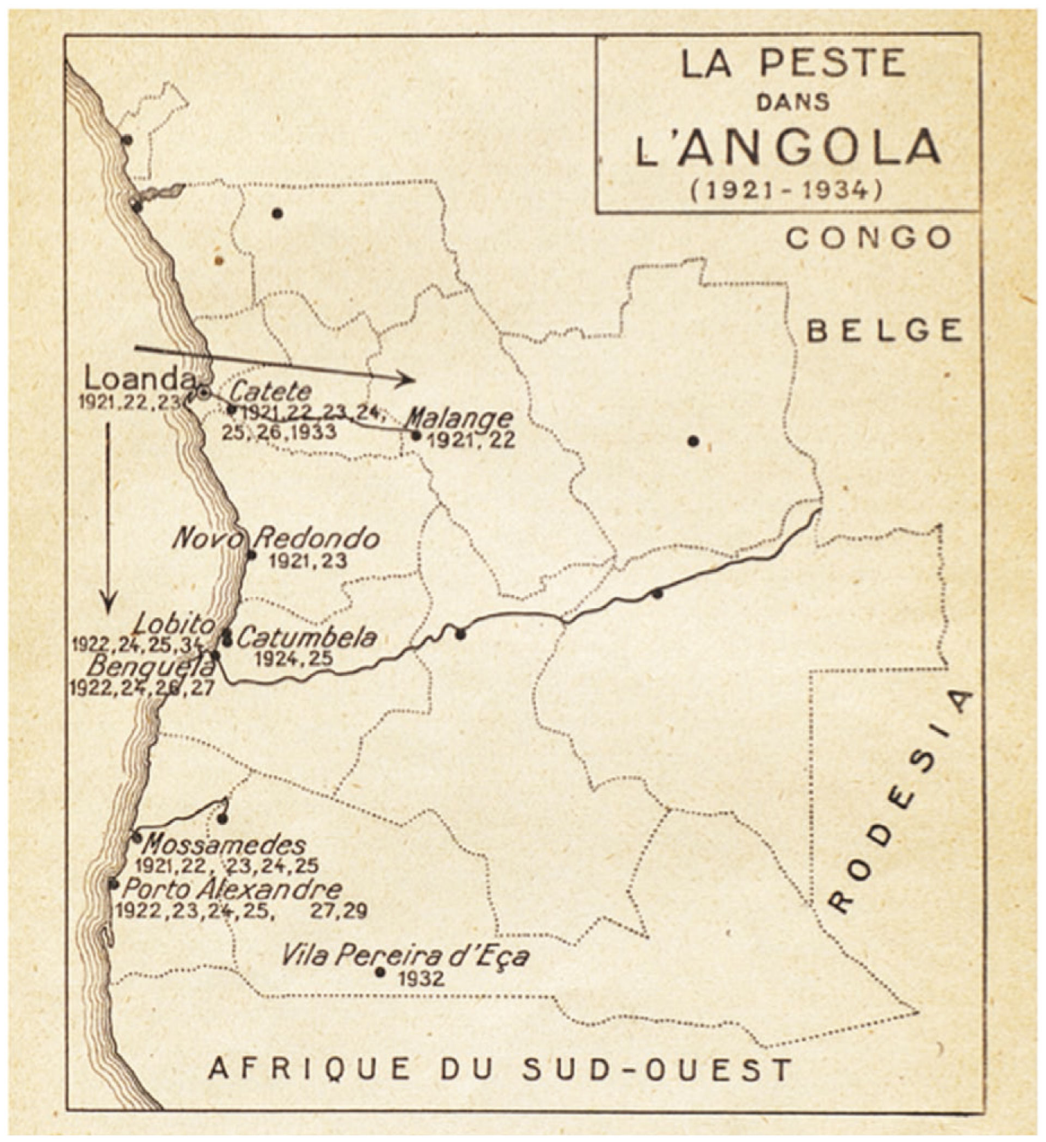
“La peste en Angola (1921-1934).” This map of Angola drawn at the Office International Hygiène Publique shows the main outbreaks in Angola from 1921 to 1934. From Ricardo Jorge, *La peste en Angola* (Paris: Office International d’Hygiène Publique, 1935), 3.

**Figure 4:**
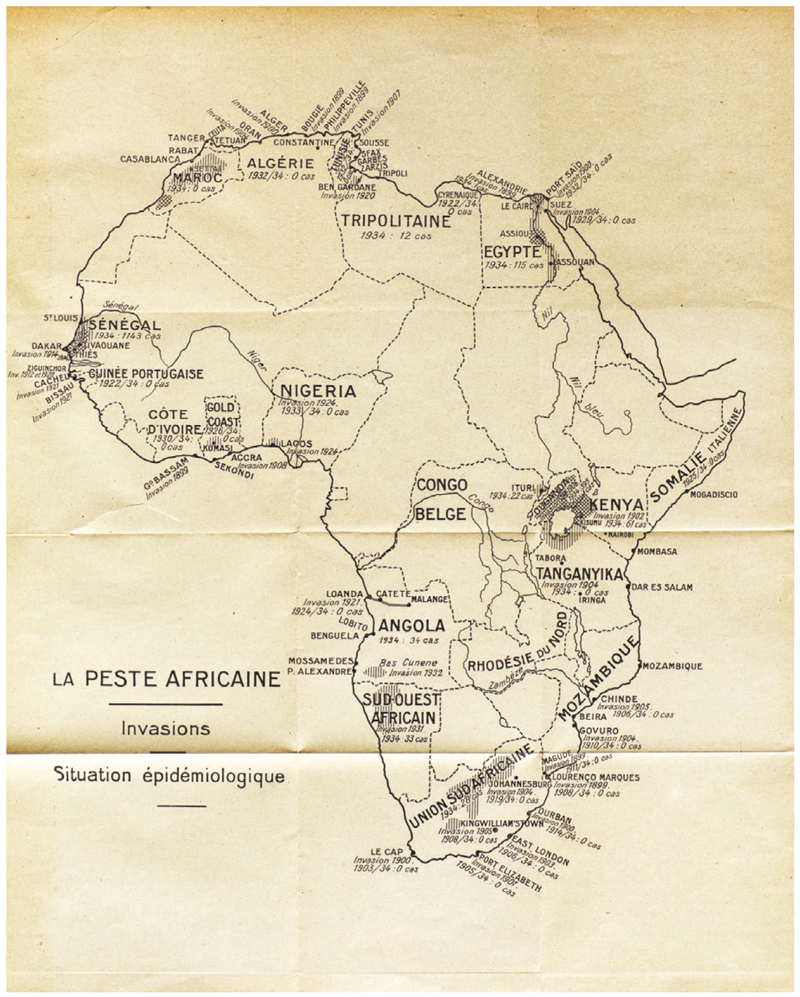
“La Peste Africaine/Invasions/Situation Epidemiologique (1935).” This map of Africa shows plague invasions from 1899 to 1932 and the epidemiological situation in 1934. From Ricardo Jorge, “La peste africaine,” *Bulletin Mensuel de l’Office International d’Hygiène Publique* 27: 9 (1935): 30-31.

After receiving the conclusions of the conference, the OIHP decided to sponsor a survey on plague in Africa. Ricardo Jorge, Portuguese delegate at the OIHP and a renowned plague expert, was charged with synthesizing reports forwarded by African countries and colonies.^99^ In Jorge’s synthesis, published in September 1935, one of the main visual devices was a map of Africa entitled “La Peste Africaine/Invasions/Situation Épidémiologique” (see Figures 4 and 3).^100^ The map is dotted with several plague outbreaks, usually the first occurred in a given country, all of them labelled as “invasion,” followed by the date it occurred (see Figure 4). According to Jorge, all these plague invasions were provoked by “domestic rats,” except two: the 1931 invasion of SWA and the 1932 invasion of the Baixo Cunene. As explained by Jorge, SWA “was invaded by huge groups of arvicolid rodents. The wave crossed through the Ovamboland and reached the southern border of Angola, which is a geological prolongation of the veld.”^101^ According to Jorge, the rodents implicated in the Ovamboland and Baixo Cunene invasions were those observed in South Africa: gerbils and multimammate mice.^102^ The rodent migration coming from South Africa, and the consequent arrival of plague in Ovamboland and Angola, led Jorge to conclude “that the extreme southern zone of Angola is part now of the great sylvatic area of the South African Union.”^103^

Jorge’s report globalized both the knowledge crafted at the border and the alleged problem posed by wild rodents, giving the OIHP’s official stamp to disputed and controversial matters. Nonetheless, Jorge also added a new layer to Fourie’s theory, stating that gerbils not only migrated, but also *invaded* Ovamboland and Angola, which ultimately enabled plague to *invade* these regions. It would be a stretch to affirm that Jorge’s categorization depicted a modern species invasiveness framework, as his report did not provide enough evidence as to whether he believed the gerbils responsible for bringing plague to Ovamboland/ the Baixo Cunene were complete foreigners to the region, or if their migrations, or invasions, were regular events. Nevertheless, the idea that certain gerbil species were not native to the region quickly gained ground among Portuguese doctors.

## Routinization and Collaboration

Returning to the border between Angola and SWA, by July 1932 plague management shifted to routinization on both sides. In Ovamboland, it took the form of periodic inspections by Schetteler, disinfestations of homesteads, treatment of patients, rodent surveys to find evidence of plague, and the collection of rodent specimens for museums and educational services.^104^ However, such measures, hastily formulated by Fourie, proved difficult to implement. According to van Niekerk, Fourie’s disinfestation recommendations could not easily be enforced because living conditions in Ovambo *eumbos*, which comprised of wood, clay, mud, and thatch huts, as well as open air enclosures, were antithetical to rat-proofing techniques developed in cities. So-called “kraal rodents” were often too difficult to remove from the *eumbos* they had invaded. According to van Niekerk, combatting plague in the region was extremely difficult because, “The veld rodent offers no solution as its extermination is impossible. Thus an attempt must be made to ‘rat proof’ the native kraal, if such a term can conceivably be applied to a habitation of its description. What it really amounts to is that the head of each and every kraal must make it his duty to see that a constant war is waged on the rodent in and around his kraal.”^105^ Despite such colonial grumbling, what little evidence exists suggests that Ovambo groups were mostly in support of fighting *Y. pestis* in Ovamboland, and of “kraal rodents” into their *eumbos*. Fourie and van Niekerk both thought that Ovambo support or opposition was polarized along Christian / independent religious lines. Many groups had long been exposed to White doctors through the presence of Finnish medical missionaries, and some had converted to Christianity.^106^ There appears to have been little opposition to vaccination and treatment amongst these groups. According to Fourie and van Niekerk, those who practiced Indigenous religions allegedly did not cooperate with surveillance systems or go to settler doctors for treatment, preferring to consult their own healers.^107^

**Figure 5:**
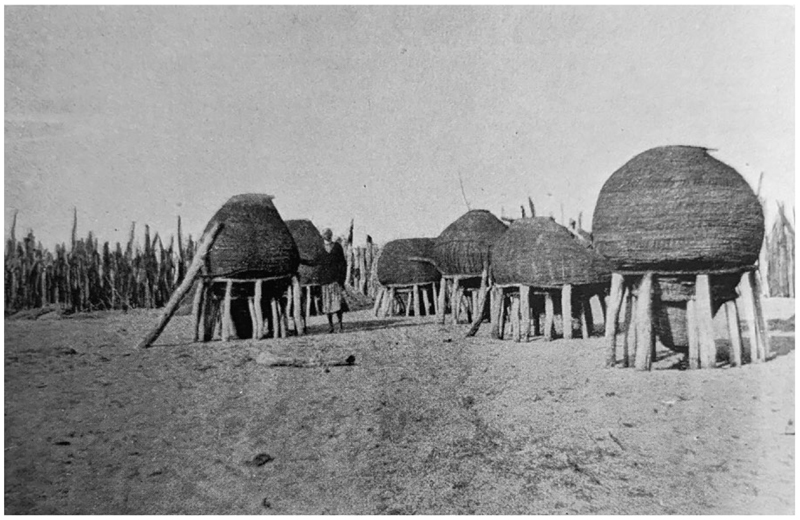
Ovambo raised grain baskets, photographed in the 1920s. From C.H.L. Hahn, L. Fourie, and H. Vedder, *The Native Tribes of South West Africa* (Cape Town: Cape Times Limited, 1928), insert at page 28.

Disease treatment aside, rodent disinfestation seems to have been an uncontroversial intervention. In his dozens of reports, Schettler never complained about a lack of cooperation of any Ovambo group. Between May 1932 and November 1933, he and his team of African laborers disinfested a total of approximately 1,277 huts, and consistently relied upon Ovambo people to lead them to places of rodent infestation or plague.^108^ There are several potential reasons behind this energetic collaboration here. On the one hand, having witnessed or heard stories of the shocking South African aerial bombardment of Iipumbu yaShilongo’s compound in August 1932, Ovambo groups may have been fearful of contravening colonial instructions from this point onwards.^109^ On the other hand, it is possible that Ovambo groups saw their own pest-control as well as medical goals and the colonial state’s anti-plague goals as aligned. Rodent-collecting expeditions conducted by Shortridge in the 1920s revealed that Ovambo people knew the rodents of the area extremely well and had their own names for almost all of the species he collected.^110^ Many of these animals were shy and lived underground or in trees, indicating that there was extensive Indigenous rodent catching in the region, likely for pest control, for eating, or for both. Ovambo people grew large quantities of millet and sorghum as staples, and stored these in raised baskets which may have attracted unwelcome rodents (see Figure 5).^111^ Fourie himself remarked that Ovambo youths were “most expert at catching rats,” and that Ovambo people referred to the multimammate mouse as the “kraal mouse” and complained of it causing “considerable damage” in “gnawing holes into the corn-baskets.”^112^ Ovambo people also lived with dogs, who likely attacked rodents.^113^ By 1933, van Niekerk even reported that certain homesteads were inventing “ingenious home-made” rodent traps to crush the animals with rocks.^114^

It is also possible that there was considerable common ground between colonial understandings of the etiology and epidemiology of plague and Ovambo medical thought. According to Davies, Ovambo people had medical concepts for “large-scale illness, such as an epidemic,” as well as infection, contamination, and contagion.^115^ There were also ideas in circulation that humans could contract diseases from animals. Many Ovambo people thought that anthrax could be caught from eating the meat of infected cattle, while one mental disorder was thought to be contracted from birds flying above.^116^ Additionally, “a dead mouse found in a household” was considered an “omen of death,” indicating a shared fear of dying rodents.^117^ Fourie reported that Ovambo people had even correlated rodents and plague, referring to the disease as “‘mouse’ disease.”^118^ Whether or not these concepts and fears were lifted from settler medical vocabularies, this nevertheless indicates some commonality between settler and Ovambo etiological and epidemiological understandings of this disease.^119^ As such, they evidently did not wish to live side by side with rodents and were committed to repelling their invasions into homes and granaries.

**Figure 6:**
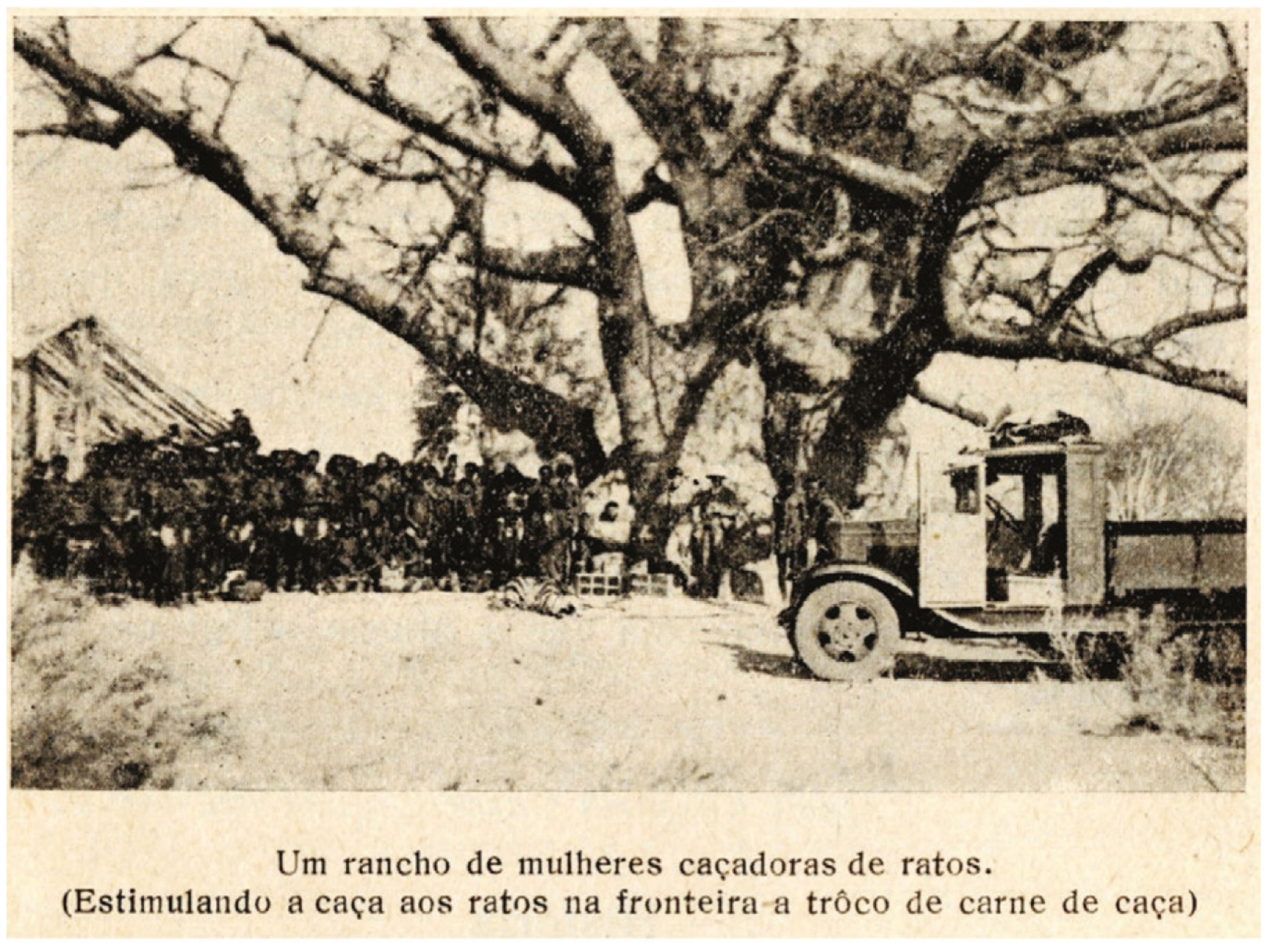
The original caption translated into English reads, “A ranch/kraal of women rat-catchers (stimulating rat-catching at the border in exchange for game).” From Francisco Venâncio da Silva, Serviço Permanente de Prevenção e Combate à Peste Bubônica no Sul de Angola/ Relatório 1933 (Lisboa: Divisão de Publicaçōes e Biblioteca, Agência Geral das Colônias, 1936), 16.

In the Baixo Cunene, Silva now believed that “propaganda and the education of the indigenous [sic] [were] the best prophylactic weapon,” rather than coercive measures, such as sanitary cordons.^120^ Accordingly, in September 1932 the Missão published a pamphlet in Portuguese and Oshikwanyama explaining the links between rodents and plague, and inviting the local population to kill rodents.^121^ The Missão and later the Serviço also chose direct teaching, which was central in a region where the great majority of the population was illiterate. Silva and his assistants traversed the Baixo Cunene region asking Indigenous people to make their dwellings rodent-proof, chase rodents from their villages, and protect the rodents’ natural enemies, such as snakes and birds, in an effort to disrupt human-rodent interactions (see Figure 6). In addition, the local population was urged to inform the Serviço of any case of plague among humans or any epizootic among wild rodents. The Serviço would then come to the village in question, vaccinate its population against plague, and pump cyanide gas into wild rodent holes in the fields nearby.^122^ As Antonio Damas Mora enthusiastically wrote to Jorge, “no rat burrow escapes the disinfection.”^123^ Hence, not only did blaming gerbils serve to bring SWA, South Africa, and Angola into collaboration, it also drew the cooperation and scientific involvement of Ovambo people on both sides of the border, especially the Oukwanyama.

## Rodent Collection and the Formalization of an Invasive Species Framework

Plague in the Baixo Cunene pitted Silva against an unknown element, the local wild rodents, and thus he believed that gathering up-to-date information about them was essential to his work. However, he thought that the existing information written by Portuguese zoologists on wild rodents of this region was outdated, and thus turned to South African experts, starting an epistolic exchange with Fourie.^124^ Silva sent Fourie several rodents he had captured in the Baixo Cunene and in exchange, Fourie sent Silva papers regarding plague in South Africa, his report on the Ovamboland outbreak, and two articles by Oldfield Thomas on rodents from Ovamboland, the Cunene River, and the Gobabis district in Eastern Damaraland (also in SWA) collected during Shortridge’s third expedition in 1924.^125^ Given the difficulties in obtaining information about the Baixo Cunene ecology, Silva also sought help from the “natives,” who he considered his best “teachers” about the Baixo Cunene rodent fauna.^126^ The Indigenous populations helped Silva to capture rodents, to understand their behavior, and to establish their local names.^127^ Silva also corresponded with van Niekerk, comparing their collections, exchanging specimens, and determining their scientific names.^128^ This continuous collection of rodents made the animals move once again, this time not as vital agents with the capacity to spread disease across borders but rather as taxidermized remains, circulated for zoology and for medical education.^129^

Helped by the different peoples of the Baixo Cunene, South West African, South African and Swiss experts visiting the Baixo Cunene, and mobilizing their different knowledges, Silva began classifying the local fauna of rodents and establishing possible links between them and past and future plague outbreaks.^130^ Silva developed in more detail what Hinsbeeck had suspected in April 1932 – that the South African plague-carrying *Desmodillus auricularis pudicus* was new to the region. In a list of rodents and other animals sent to the Bocage Museum in Lisbon in 1934, Silva noted that this rodent was “known by the English as the Namaqua Gerbille, [and] of recent immigration to neighboring Ovamboland, where it was caught very close to our border. It has not yet been found in Angola, but it will be, inevitably.”^131^ This new prophecy came to fruition a few years later. In a similar list compiled in 1939, when plague seemed to have disappeared from the Baixo Cunene, Silva confirmed that *Desmodillus auricularis pudicus* “did not exist in Angola until 1932. From a recent migration, it has no name among the indigenous, and only exists in the area very close to the border. It was considered for a while not susceptible to the experimental inoculation [of plague], but it was by this animal that the epizootic wave reached [*atingiu*] the Ovamboland.”^132^

Silva’s work signaled an important epistemological shift in the framing of the plague outbreak in Ovamboland and the Baixo Cunene. If since 1932 Portuguese doctors agreed with Fourie and understood these episodes in terms of an invasion of a new disease into the region, carried by numerous species of migratory gerbils sometimes framed as invaders, in 1939 Silva added a new layer to make sense of the outbreaks in Ovamboland and the Baixo Cunene. He described these episodes in terms of what we could today call an invasive species framework. He indicted a *single* alien species to the ecology of the Baixo Cunene, *Desmodillus auricularis pudicus*, for bringing the other “invader,” the plague bacillus, to the region.

## Conclusion

By focusing on the plague outbreaks in Ovamboland and the Baixo Cunene starting in 1932, this article has illuminated several iterations of the invasiveness framework, showing how it justified sanitary actions against human and non-human invaders, and how these invaders brought different human actors into conflict and collaboration. From the outset of the affair, questions of health were central to the emergence of an invasiveness framework. Not only did plague draw attention to Southern African gerbil migrations in the first place, but also the plague bacillus was prominently framed as an invader: it was considered the ultimate alien object to a region distant from ports and colonial cities. However, the element responsible for bringing the bacillus to Ovamboland and the Baixo Cunene changed over the 1930s. Firstly, Hinsbeeck blamed grain from South Africa for importing the bacillus into region, after which it took on an invasive character through the agency of Indigenous rodents. Meanwhile, the Portuguese in particular framed Ovambo people themselves as potential drivers of plague invasion into the south of Angola, resulting in a cordon sanitaire at the border. Thanks to Fourie and the support of Portuguese doctors in Angola and Portugal, this framework changed: gerbils, framed as migrants or invaders, were blamed for spreading the disease across international borders and became the target of sanitary actions. Finally, in 1939, a single gerbil species – *Desmodillus auricularis pudicus –* long suspected of being foreign was framed by Silva as alien to the ecology of the Baixo Cunene and Ovamboland and responsible for the entire outbreak of plague. At each stage, the perceived activities of rodents themselves, along with colonial conceptualizations of pathological rodent agency, brought diverse human groups, empires, and international organizations together, producing new forms of knowledge about plague, rodents, and disease ecology.

These three ways of conceiving the outbreaks reveal several distinct medical frameworks. The first one, blaming grain and Ovambo people, echoes a contagionist reasoning, with quarantine and *cordons sanitaires* as its ultimate expression. With a long history of fighting epidemic diseases such as yellow fever, cholera, and plague, this framework usually understood humans, especially those perceived as “dangerous” races and classes, and human-made objects, as responsible for facilitating the spread of diseases across international borders, either because they could directly transmit the disease or because they were carrying the vector of the disease, the flea in the case of plague.^133^ The second one could be considered a zoonotic reasoning, framing disease mainly as a problem of animals, and therefore of a pathological “nature,” to be separated from humans through routinised killing and infrastructural alteration.^134^ The third is an ecologically-oriented derivation of the latter, which has gained recent prominence under the emerging infectious diseases and One Health frameworks. Outbreaks among humans are not necessarily linked to animals in general, but to certain alien species. In their “original” ecosystem, these species are not necessarily a threat, as “balance” exists between host and parasites. The problem starts when this balance is shaken by human or non-human agency and other ecosystems are brought in contact with the affected one. Therefore, a fight against diseases would not, theoretically, be a generalized war against nature, but would target the invasive species whether animal, insect, or microorganism, while trying to protect animals and humans.^135^ This said, it would be unfruitful to seek in Ovamboland/ the Baixo Cunene events a forgotten precursor of modern medical reasoning simply because it oriented towards ecological explanations. Instead, this article has made a case for more complex and rich global histories of disease ecology. Starting from a singular event, microscopically examined, we have reconstructed multiple political dynamics and forms of medical reasoning at local, national, imperial, and global scales.

## Figures and Tables

**Figure 1:**
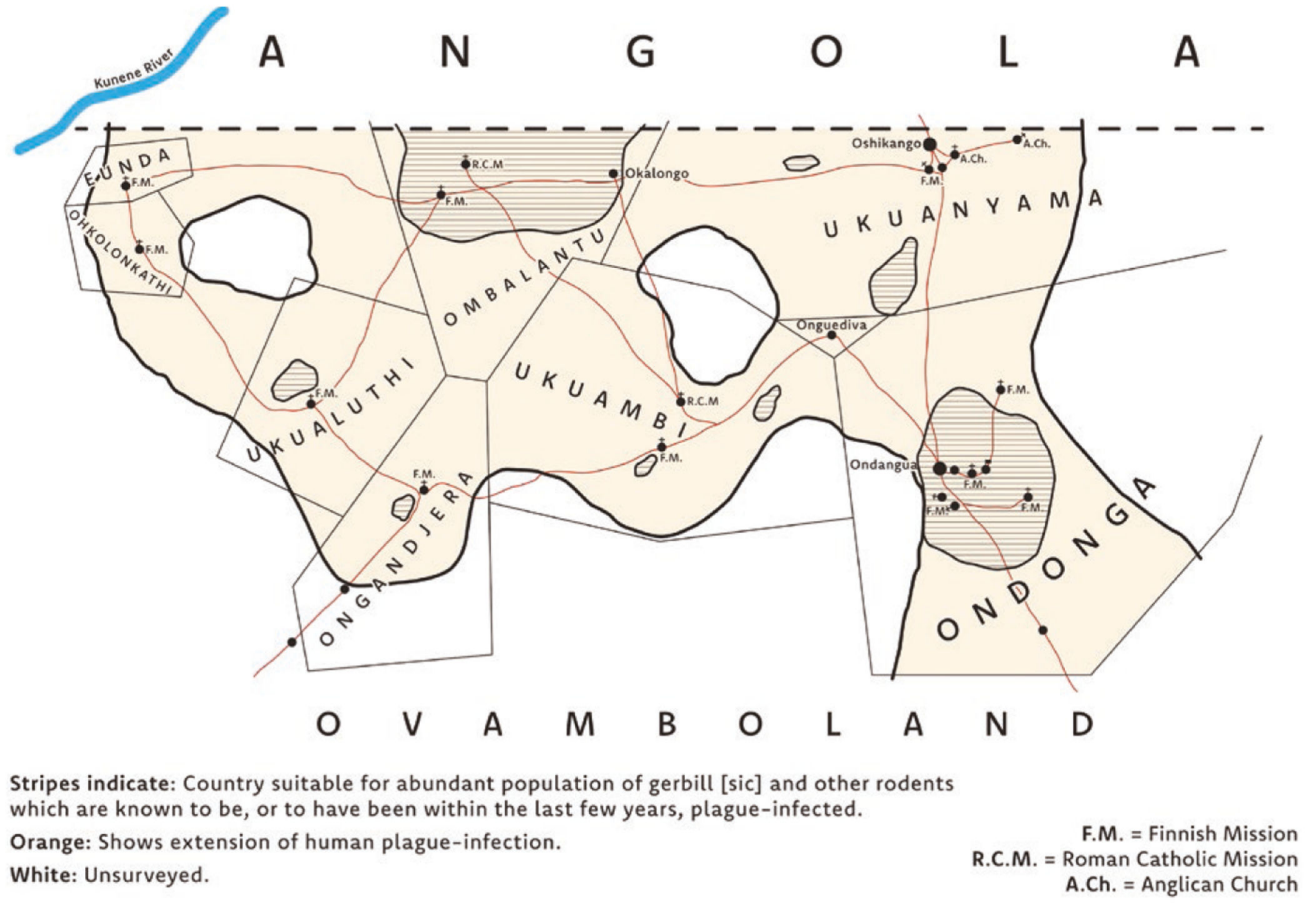
Plague in Ovamboland, 1932. Created by Leo Mewse, Print & Design, University of St Andrews, adapted from a map likely drawn by K. Schettler. The original map is appended to K. Schettler, “Monthly Report: October 1932,” dated 31 October 1932 in NAN, NAO 34, File 26/27, “Plague.”

## Footnotes

1 Michiel van Niekerk to Chief Medical Officer, 10 January 1933, National Archives of Namibia (NAN), NAO 34, File 26/7; “Memorandum on the Plague Situation, Ovamboland,” NAO 34, File 26/7. The author of this memorandum was not listed, but it is appended to van Niekerk’s letter of 10 January 1933. He is almost certainly its author.

2 Sanitary officials assumed that plague would either enter the country via the ports of Luderitz or Walvis Bay, or via rail from South Africa. See Memorandum, likely 1925, NAO 34, File 26/27. For the threat posed by Angolan ports see NAO 37, File 26/9.

3 *Yersinia pestis* is the modern name for this microorganism. It was then called *Bacillus pestis* or *Bacterium pestis* in southern Africa.

4 M. van Niekerk to Chief Medical Officer, 10 January 1933, NAN, NAO 34, File 26/7.

5 Ibid. See also L. Fourie, “Plague – Ovamboland Report,” 8 June 1932, NAN, NAO 34, File 26/7; Medical Officer to the Administration to Native Commissioner Ovamboland, 11 February 1932, NAN, SWAA 1695, A331/4.

6 Antonio Damas Mora, “La peste dans l’Angola,” *Bulletin Trimestriel de l’Organisation d’Hygiène* II: 1 (1933): 99–105; Ministério das Colônias/Gabinete do Ministro, “Decreto n. 21:866,” *Diário do Governo* I Série, no. 270 (17 November 1932): 2231–2233; Francisco Venâncio da Silva, *Serviço Permanente de Prevenção e Combate à Peste Bubônica no Sul de Angola/ Relatório 1933* (Lisboa: Divisão de Publicaçōes e Biblioteca. Agência Geral das Colônias, 1936).

7 Damas Mora, “La peste dans l’Angola,” 104; Francisco Venâncio da Silva, “Relatório sobre a peste selvática em Angola. Elaborado a pedido do Prof. Dr. Ricardo Jorge” (25 June 1935), Espólio 18, caixa 18, maço 5, Biblioteca Nacional de Portugal - Reservados; Ricardo Jorge, “La peste africaine,” *Bulletin Mensuel de l’Office International d’Hygiène Publique* 27: 9 (1935): 25.

8 Francisco Venâncio da Silva, “Peste selvática, problema sempre latente em Angola,” *Anais do Instituto de Medicina Tropical* 10: 3 (1953): 976.

9 Michiel van Niekerk to Chief Medical Officer, 10 January 1933, NAN, NAO 34, File 26/7; L. Ribeiro, “Developments in Endemic Plague in Angola from 1932 Onwards,” *Quarterly Bulletin of the Health Organisation* 5: 3 (1936): 108–109.

10 South Africans also referred to this disease as “plague amongst the veld rodents” or “plague on the veld.” See Jules Skotnes-Brown, *Segregated Species: Pests, Knowledge, and Boundaries in South Africa, 1910s-40s* (Baltimore: Johns Hopkins University Press, 2024), chapter 4. On sylvatic plague, see Ricardo Jorge, “Les faunes régionales des rongeurs et des puces dans leurs rapports avec la peste (deuxième partie),” *Bulletin Mensuel de l’Office International d’Hygiène Publique* 19: 9 (1927): 1257–1288; Matheus Alves Duarte da Silva, “Between Deserts and Jungles: The Emergence and Circulation of Sylvatic Plague (1920-1950),” *Medical Anthropology* 42 (2023): 325-339.

11 Kapil Raj, *Relocating Modern Science: Circulation and the Construction of Knowledge in South Asia and Europe, 1650 - 1900* (Basingstoke: Palgrave Macmillan, 2010); Sarah Easterby-Smith, “Recalcitrant Seeds: Material Culture and the Global History of Science,” *Past & Present* 242 (2019): 215–242; Matheus Alves Duarte da Silva, “Quand la peste connectait le monde: production et circulation de savoirs microbiologiques entre Brésil, Inde et France (1894-1922)” (Thèse de Doctorat, Paris, École des Hautes Études en Sciences Sociales, 2020).

12 For the concept of an “animal trace,” see Etienne Benson, “Animal Writes: Historiography, Disciplinarity, and the Animal Trace,” in *Making Animal Meaning*, ed. Linda Kalof and Georgina Montgomery (East Lansing: Michigan State University Press, 2011), 3–16. For “pest” animal presences in archives see Kaori Nagai, “Vermin Writing,” *Journal for Maritime Research* 22 (2020): 59–74; Skotnes-Brown, *Segregated Species*, Introduction.

13 Molly McCullers, “Betwixt and Between Colony and Nation-State: Liminality, Decolonization, and the South West Africa Mandate,” *American Historical Review* 124 (2019): 1704–1708.

14 Patricia Margaret Hayes, “A History of the Ovambo of Namibia, c 1880-1935” (PhD diss., University of Cambridge, United Kingdom, 1992).

15 Gwyneth Davies, “The Medical Culture of the Ovambo of Southern Angola and Northern Namibia” (PhD diss., University of Kent, United Kingdom, 1993), 23.

16 Alicia P. Yon, “Planning for Urban Growth: With Special Reference to Windhoek, Namibia” (MA thesis, University of Tasmania, Australia, 1999), 128.

17 Reductively referred to by colonial officials as “kraals.” Hayes, “A History of the Ovambo of Namibia, c 1880-1935”; Emmanuel Kreike, *Scorched Earth: Environmental Warfare as a Crime against Humanity and Nature* (Princeton: Princeton University Press, 2021); Davies, “Medical Culture of the Ovambo of Southern Angola and Northern Namibia,” 27.

18 Hayes, “History of the Ovambo of Namibia, c 1880-1935,” 16–17. Davies designates “12 culturally related peoples” in Ovamboland. Davies, “Medical Culture of the Ovambo of Southern Angola and Northern Namibia,” 23.

19 Hayes, “History of the Ovambo of Namibia, c 1880-1935,” 264–266; Alexandre Aparicio, “O último reino independente de Angola e a fixação da fronteira sul (1910-1926),” in *A África e a Instalação do Sistema Colonial*, ed. Maria Emília Madeira Santos (Lisbon: Centro de Estudos de História e Cartografia Antiga, 2000), 228; Sonia Cristina Cardoso dos Santos Silva, “O percurso histórico do estabelecimento das fronteiras em Angola,” *Fronteiras: Revista de História* 21 (2019): 138.

20 Hayes, “History of the Ovambo of Namibia, c 1880-1935,” chapters 3–5.

21 Davies, “Medical Culture of the Ovambo of Southern Angola and Northern Namibia,” 24.

22 Kreike, *Scorched Earth*, chapter 10.

23 Crews argues that Iipumbu had resisted colonial control of his kingdom at every turn since his rise to power in 1907 or 1908. The standoff with the missionaries took place because missionaries had given Iipumbu’s “social and perhaps even… biological daughter” asylum from Iipumbu’s alleged sexual abuse. In turn, Iipumbu had threatened the missionaries with retaliation for months. Davies, “Medical Culture of the Ovambo of Southern Angola and Northern Namibia,” 22, 33; Emily D. Crews, “Singing ‘The Song of Chief Iipumbu’: Christianity, Colonialism, and the Limits of Agency in North-Central Namibia,” *Religion and Theology* 25 (2018): 268.

24 Van Niekerk to Medical Officer to the Administration, 28 January 1932, NAN, NAO 34, File 26/27.

25 Medical Officer to the Administration to Administrator of South West Africa, 11 February 1932, NAN, NAO 34, File 26/27.

26 Van Niekerk to Medical Officer to the Administration, 8 March 1932, NAN, NAO 34, File 26/27.

27 Medical Officer to the Administration to Administrator, Windhoek, 28 April 1932, NAO 34, File 26/27.

28 Medical Officer to the Administration to Edward Thornton, 23 February 1932, NAN, SWAA 1695, A331/4. For more on the grain theory of plague, see Christos Lynteris, “In Search of Lost Fleas: Reconsidering Paul-Louis Simond’s Contribution to the Study of the Propagation of Plague,” *Medical History* 66 (2022): 242–263.

29 Medical Officer to the Administration to Edward Thornton, 23 February 1932, NAN, SWAA 1695, A331/4.

30 Ibid.

31 Edward North Thornton to F.C.S. Hinsbeeck, dated 17 February 1932, NAN, SWAA 1695, A331/4.

32 Johannes Hendrik Loots, “The Health Conditions of the Native Population in Ovamboland, and Its Bearing on the Development of South West Africa” (MD thesis, University of Edinburgh, 1930), 19–20.

33 Volharding, Geneva to Secretary for External Affairs, Pretoria, 19 June 1932, NAN, SWAA 1695, A331/4. See also F.F. Pienaar, Accredited Representative Geneva, to Secretary for External Affairs, Pretoria, 6 July 1932, South African National Archives Repository (Public Records of Central Government since 1910) (SAB), PM 1/2/176, PM 49/91.

34 Emphasis added. Hinsbeeck to the Administrator, 28 April 1932, NAO 34, File 26/27.

35 Damas Mora, “La peste dans l’Angola,” 99.

36 Ibid.

37 João de Almeida Souza Sacadoura to NCO, 26 February 1932, NAO 34, File 26/27.

38 Antonio Damas Mora, “La peste en Angola,” *Revista Médica de Angola* III, no. 4 (1923): 323.

39 Ibid.

40 João de Almeida Souza Sacadoura to NCO, 26 February 1932, NAO 34, File 26/27.

41 McHugh to Native Commissioner Ovamboland (NCO), 23 March 1932, NAO 34, File 26/27. João de Almeida Souza Sacadoura to NCO, 23 March 1932 NAO 34, File 26/27.

42 NCO to Secretary for SWA, 5 March 1932, NAO 34, File 26/27.

43 McHugh to NCO, 25 April 1932, NAO 34, File 26/27. The Portuguese, on the other hand, stressed that the arrival of winter and a lack of water played the central role on the dismantlement of the cordon sanitaire maintained by the troops. See Damas Mora, “La peste dans l’Angola,” 99.

44 Davies has dismissed this claim as “quite incorrect” and dismantled it in her doctoral thesis. Davies, “Medical Culture of the Ovambo of Southern Angola and Northern Namibia,” 51.

45 Loots, “Health Conditions of the Native Population in Ovamboland, and Its Bearing on the Development of South West Africa,” 6–7.

46 Davies, “Medical Culture of the Ovambo of Southern Angola and Northern Namibia,” 24.

47 Secretary for SWA to Magistrate of Luderitz, 29 January 1932, SWAA 1695, A331/4; van Niekerk to Chief Medical Officer of SWA, 10 January 1933, NAO 34, File 26/27.

48 Secretary for SWA to Health Department Cape Town, 24 March 1932, SWAA 1695, A331/4.

49 C.H.L. Hahn, L. Fourie, and H. Vedder, *The Native Tribes of South West Africa* (Cape Town: Cape Times Limited, 1928). For more on his ethnography, see Ann Wanless, “The Silence of Colonial Melancholy: The Fourie Collection of Khoisan Ethnologica Volume 1” (PhD diss., University of the Witwatersrand, Johannesburg, 2007). For his plague research see Skotnes-Brown, *Segregated Species*, chapter 4.

50 Damas Mora, “La peste dans l’Angola,” 100.

51 “ITIS - Report: Gerbilliscus (Taterona),” accessed 17 May 2023, https://www.itis.gov/servlet/SingleRpt/SingleRpt?search_topic=TSN&search_value=970538#null.

52 For animals, underground infrastructure, and zoonosis, see Genese Marie Sodikoff, “The Multispecies Infrastructure of Zoonosis,” in *The Anthropology of Epidemics*, ed. Ann Kelly, Frédéric Keck, and Christos Lynteris (London: Routledge, 2019).

53 L. Fourie, “Plague – Ovamboland Report,” 8 June 1932, NAO 34, File 26/7.

54 Ibid.

55 Ibid.

56 Ibid.

57 Ibid. This references a technique for detecting plague outbreaks developed by South Africa’s Chief Rodent Inspector William Powell. See Skotnes-Brown, *Segregated Species*, chapter 4.

58 L. Fourie, “Plague – Ovamboland Report,” 8 June 1932, NAO 34, File 26/7.

59 Ibid.

60 Ibid.

61 Ibid.

62 Ibid. Shortridge’s Ovamboland survey was the most comprehensive mammal survey of any African region in this period. Many of his contemporaries regarded it a definitive account of every mammal species in the region. The *Daily Dispatch* newspaper claimed that “it is doubtful if any single thing, large or small, with the possible exception of one or two obscure things like bats, has been overlooked.” “Mammals of South-West Africa,” *Daily Dispatch*, 1928, newspaper clipping in Amatole Museum Library, “Newspaper Cuttings and Annual Reports, 1921-1932.” Fourie was also involved in Shortridge’s surveys. Shortridge wrote that he was “one of our greatest helpers.” See Shortridge to Oldfield Thomas, 11 May 1925, Natural History Museum Archives (London), DF/ZOO/232/6/7/1010.

63 Medical Officer to the Administration to The Administrator, Windhoek, 28 April 1932, NAO 34, File 26/27.

64 L. Fourie, “Plague – Ovamboland Report,” 8 June 1932, NAO 34, File 26/7.

65 De Souza to C. Hahn, 27 August 1932, NAO 34, File 26/27. Venâncio da Silva, *Serviço Permanente de Prevenção e Combate à Peste Bubônica no Sul de Angola/ Relatório 1933*, 155.

66 Matheus Alves Duarte Da Silva, “Plague in Portugal: Between the Local and the Global,” in *Portuguese Colonial Cities*, ed. Cláudia C. Azeredo Atallah Maria Sarita Mota (Buenos Aires: Teseo, 2023), 395–420.

67 Ricardo Jorge, *A peste bubônica no Porto-1899. Seu descobrimento. Primeiros trabalhos* (Porto: Repartição de Saúde e Higiene da Câmara do Porto, 1899); Jaime Ferran, Rosendo de Grau, and Frederico Viñas y Cusi, *La peste bubónica: Memoria sobre la epidemia ocurrida en Porto en 1899* (Barcelona: Tip. Sucesor F. Sanchez, 1907); Maria Antónia Pires Almeida, “Fighting Disease and Epidemics: Ricardo Jorge and the Internationalization of Portuguese Science,” *Vesalius: Acta Internationales Historiae Medicinae* 19 (2013): 19–23.

68 The arrival of plague in Luanda was suspected to be an importation from Lisbon. See Damas Mora, “La peste en Angola,” 319.

69 Ricardo Jorge, *La peste en Angola* (Paris: Office International d’Hygiène Publique, 1935), 2-4.

70 The Baixo Cunene is almost 1,000 km away from Luanda. Ibid., 5.

71 Damas Mora, “La peste dans l’Angola,” 99.

72 “Letter from Adolpho Trindade, Portuguese Consul in Windhoek, to the Diretor Geral Dos Negócios Comerciais” Windhoek, 7 May 1932, Peste no estrangeiro. Peste bubonica em Ovamboland 10 (3) 32, Arquivo Histórico Diplomático - Portugal.

73 “Letter from F. A. Correia, Diretor Geral dos Negócios Comerciais, to the Diretor Geral das Colônias do Ocidente and the Diretor Geral da Saúde” Lisbon, 15 June 1932, Peste no estrangeiro. Peste bubonica em Ovamboland 10 (3) 32, Arquivo Histórico Diplomático - Portugal.

74 Office International d’Hygiène Publique, “Notification no 998” Paris, 17 May 1932. Peste no Estrangeiro. S13. E15. P4/82832, Arquivo Histórico Diplomático - Portugal.

75 Ibid.

76 Office International d’Hygiène Publique, “Notification no 1009” Paris, 14 June 1932, Peste no Estrangeiro. S13. E15. P4/82832, Arquivo Histórico Diplomático - Portugal.

77 On prophetization and medical reasoning, see Carlo Caduff, “Pandemic Prophecy, or How to Have Faith in Reason,” *Current Anthropology* 55 (2014): 296–315.

78 Ministério das Colônias/Gabinete do Ministro, “Decreto n. 21:866.” The Missão is named slightly differently in other official documents. See Missão de Defesa e Combate à Peste Bubónica no Sul de Angola, *Peste bubónica: noçōes gerais sóbre a sintomatologia e epidemiologia* (Huila: Colónia de Angola, 1932).

79 Venâncio da Silva, *Serviço Permanente de Prevenção e Combate à Peste Bubônica no Sul de Angola/ Relatório 1933*, 13–14.

80 Ibid., 74–75.

81 Davies, “Medical Culture of the Ovambo of Southern Angola and Northern Namibia,” 32.

82 On the conquest of the Baixo Cunene, see Aparicio, “O último reino independente de Angola e a fixação da fronteira sul (1910-1926),” 223–224.

83 On the border, see ibid.; Cristina Udelsmann Rodrigues, “The Kwanhama Partitioned by the Border and the Angolan Perspective of Cross-Border Identity,” *African Studies* 76 (2017): 423–443; Silva, “O percurso histórico do estabelecimento das fronteiras em Angola.”

84 Raimundo Serrão, “Introdução,” in Venâncio da Silva, *Serviço Permanente de Prevenção e Combate à Peste Bubônica no Sul de Angola/ Relatório 1933*, 7–8.

85 Ministério das Colônias/Gabinete do Ministro, “Decreto n. 21:866”; Venâncio da Silva, *Serviço Permanente de Prevenção e Combate à Peste Bubônica no Sul de Angola/ Relatório 1933*, 14–15.

86 Venâncio da Silva, *Serviço Permanente de Prevenção e Combate à Peste Bubônica no Sul de Angola/ Relatório 1933*, 12–15.

87 Ibid., 11, 20.

88 Hahn to Secretary for SWA, 30 June 1932, NAO 34, File 26/27.

89 Ibid.

90 Damas Mora, “La peste dans l’Angola,” 104.

91 Missão de Defesa e Combate à Peste Bubónica no Sul de Angola, *Peste bubónica: noçōes gerais sóbre a sintomatologia e epidemiologia* (Huila: Colónia de Angola, 1932).

92 Damas Mora, “La peste dans l’Angola,” 105; Ministério das Colônias/Gabinete do Ministro, “Decreto n. 21:866.” In some official documents, the word “epidemia” is removed from the name of the “Serviço.” See Venâncio da Silva, *Serviço Permanente de Prevenção e Combate à Peste Bubônica no Sul de Angola/ Relatório 1933*.

93 “Letter from H. Ribeiro to Karl Meyer” Lisbon, 11 April 1973, Karl Meyer’s papers, additions, 83.45 cz, Carton 83, Folder 45, Angola, Brancroft Library.

94 This international health conference drew participants from the League of Nations Health Committee, British India as an observer, the Rockefeller Foundation, and several British and Portuguese colonies. The conference’s primary subject was the prevention of airplanes spreading diseases such as yellow fever, but plague was also discussed. See Office International d’Hygiène Publique, *Session ordinaire d’octobre 1932 du Comité Permanent de l’Office International d’Hygiène Publique: procès-verbaux des séances* (Paris: Imprimerie Nationale, 1933), 50.

95 Edward North Thornton to Minister of Public Health, 15 December 1932, SAB, GES 2988, LN1. It is not clear why heavy rain was such a concern to Thornton. Perhaps he thought this would flush gerbils from their burrows.

96 Thornton to Minister of Public Health, 15 December 1932, SAB, GES 2988, LN1.

97 S.W.T. Lee, Rapporteur, “Report on the position in regard to, and measures employed to combat, plague in the Union of S. Africa, the Mandated Territory of S.W. Africa, Angola, the East African Colonies and Protectorates, Bechuanaland and other African countries represented at the Conference,” SAB, GES 2988, LN1.

98 Anonymous, “La Conférence du Cap: rapport de la conférence internationale des représentants des services d’hygiène de certains territoires africains et de l’Inde Britannique, tenue au Cap du 15 au 25 Novembre 1932. Part II. Rapport sur la peste,” *Bulletin Trimestriel de l’Organisation d’Hygiène* II, no. 1 (March 1933): 69–71.

99 Jorge, “La peste africaine.” In 1927, Ricardo Jorge synthesised for the OIHP a global survey on rats, rodents, and plague, in which he coined the concept of sylvatic plague. See Silva, “Between Deserts and Jungles: The Emergence and Circulation of Sylvatic Plague (1920-1950).”

100 Jorge, “La peste africaine,” 30–31.

101 Ibid., 48.

102 Ibid.

103 Ibid.

104 NAN, NAO 35, Files 26/7-26/9. For specimen collecting see Director of Education to Native Commissioner of Ovamboland, 23 August 1932, NAO 34, File 26/7; Native Commissioner Ovamboland to Secretary for South West Africa, 20 June 1933, NAO 34, File 26/27; L. Fourie, “Plague – Ovamboland Report,” 8 June 1932, NAO 34, File 26/7.

105 “Memorandum on the Plague Situation, Ovamboland,” NAO 34, File 26/7.

106 Davies’s doctoral thesis on the medical culture of Ovambo peoples lends support to Fourie’s view. Although anyone was welcome at missionary clinics, it was typically Christian converts who tended to go. This was because, on the one hand, “missionary medical centres were regarded with a certain amount of fear and distrust.” On the other hand, many Ovambo groups were simply too far away from medical centers to benefit from them which “in part accounts for the prevalence of converts at the clinics, as they were obviously living at or near to the missions concerned.” Davies, “Medical Culture of the Ovambo of Southern Angola and Northern Namibia,” 43–45, quotes on 43 and 45.

107 L. Fourie, “Plague – Ovamboland Report,” 8 June 1932, NAN, NAO 34, File 26/7; van Niekerk to Chief Medical Officer, 10 January 1933, NAO 34, File 26/27.

108 Monthly Reports of the Rodent Inspector, Ovamboland, 1932-33, NAO 34, File 26/27; K. Schettler to NCO, 31 August 1933, NAO 34, File 26/27.

109 Crews, “Singing ‘The Song of Chief Iipumbu,’” 268.

110 “Shortridges Notes on Mammals of South West Africa,” Subfile: “Field Notes on Rodents in South-West Africa by G.C. Shortridge,” Shortridge’s Notes on Mammals of South West Africa, Amathole Museum Archives.

111 Davies, “Medical Culture of the Ovambo of Southern Angola and Northern Namibia,” 51–52.

112 L. Fourie, “Plague – Ovamboland Report,” 8 June 1932, NAO 34, File 26/7.

113 Ibid.

114 Van Niekerk to Medical Officer to the Administration, 19 January 1934, NAO 34, File 26/27.

115 Davies, “Medical Culture of the Ovambo of Southern Angola and Northern Namibia,” 73–74, quote on 73.

116 Davies notes that in cases of the disease “oudu odila,” children were thought to “catch convulsions from the [Onghombe] bird as it flies over them.” Ibid., 94–95, quote on 94.

117 Ibid., 92.

118 L. Fourie, “Plague – Ovamboland Report,” 8 June 1932, NAO 34, File 26/7. Davies’s thesis again lends support to Fourie’s view. She states that Ovambo peoples use the term “uuva uombuku” for bubonic plague, literally meaning “the disease of mice.” Davies, “Medical Culture of the Ovambo of Southern Angola and Northern Namibia,” 65.

119 Davies notes several medical loan words from German in Ovambo vocabularies, stating that “it is not an easy task to distinguish the new [words] from the original.” Davies, “Medical Culture of the Ovambo of Southern Angola and Northern Namibia,” 74.

120 Venâncio da Silva, *Serviço Permanente de Prevenção e Combate à Peste Bubônica no Sul de Angola/ Relatório 1933*, 154.

121 Missão de Defesa e Combate à Peste Bubónica no Sul de Angola, *Peste bubónica: noçōes gerais sóbre a sintomatologia e epidemiologia*.

122 Venâncio da Silva, *Serviço Permanente de Prevenção e Combate à Peste Bubônica no Sul de Angola/ Relatório 1933*, 149–153.

123 Antonio Damas Mora, “Letter from Antonio Damas Mora to Ricardo Jorge” Luanda, 15 May 1933, Cx 32, Biblioteca Nacional de Portugal - Reservados.

124 Venâncio da Silva, *Serviço Permanente de Prevenção e Combate à Peste Bubônica no Sul de Angola/ Relatório 1933*, 135, 137.

125 Ibid., 136–137. On these two articles, see Oldfield Thomas, “On Mammals from Ovamboland and the Cunene River, Obtained during Capt. Shortridge’s Third Percy Sladen and Kaffrarian Museum Expedition into South-West Africa,” *Proceedings of the Zoological Society of London* 96 (1926): 285–312; Oldfield Thomas, “On Mammals from the Gobabis District, Eastern Damaraland, South-West Africa, Obtained during Captain Shortridge’s Fourth Percy Sladen and Kaffrarian Museum Expedition. With Field-Notes by the Collector,” *Proceedings of the Zoological Society of London* 97 (1927): 371–398.

126 Venâncio da Silva, *Serviço Permanente de Prevenção e Combate à Peste Bubônica no Sul de Angola/ Relatório 1933*, 135-136.

127 Ibid.

128 Ibid., 136.

129 See for example, Director of Education to NCO, 23 August 1932, NAO 34, File 26/7; NCO to Secretary for SWA, 21 June 1933, NAO 34, File 26/7.

130 Venâncio da Silva, *Serviço Permanente de Prevenção e Combate à Peste Bubônica no Sul de Angola/ Relatório 1933*, 135-148. A first list of rodents from the Baixo Cunene compiled by Silva by the end of 1932 was presented by Damas Mora, “La peste dans l’Angola,” 102.

131 Venâncio da Silva, *Serviço Permanente de Prevenção e Combate à Peste Bubônica no Sul de Angola/ Relatório 1933*, 143.

132 Francisco Venâncio da Silva, *Animais bravos nas suas relaçöes com os problemas sanitários do Sul de Angola* (Luanda: Imprensa Nacional, 1939), 25.

133 Mark Harrison, “Disease, Diplomacy and International Commerce: The Origins of International Sanitary Regulation in the Nineteenth Century,” *Journal of Global History* 1 (2006): 197–217; Sylvia Chiffoleau, *Genèse de la santé publique internationale: de la peste d’Orient à l’OMS* (Beyrouth: Ifpo - Institut français du Proche-Orient, 2012); Mark Harrison, *Contagion: How Commerce Has Spread Disease* (New Haven: Yale University Press, 2013).

134 Christos Lynteris, “Zoonotic Diagrams: Mastering and Unsettling Human-Animal Relations,” *Journal of the Royal Anthropological Institute* 23 (2017): 463–485; Jules Skotnes-Brown, “Scurrying Seafarers: Shipboard Rats, Plague, and the Land/Sea Border,” *Journal of Global History* 18 (2023): 108-130.

135 Andrew Lakoff, *Unprepared: Global Health in a Time of Emergency* (Berkeley: University of California Press, 2017); Frédéric Keck, *Avian Reservoirs: Virus Hunters and Birdwatchers in Chinese Sentinel Posts* (Durham: Duke University Press, 2020).

